# The effects of green cardamom supplementation on blood pressure and endothelium function in type 2 diabetic patients: A study protocol for a randomized controlled clinical trial

**DOI:** 10.1097/MD.0000000000011005

**Published:** 2020-05-01

**Authors:** Shohreh Ghazi Zahedi, Fariba Koohdani, Mostafa Qorbani, Fereydoun Siassi, Ali Keshavarz, Ensieh Nasli-Esfahani, Mohadeseh Aghasi, Hoorieh Khoshamal, Gity Sotoudeh

**Affiliations:** a Department of Community Nutrition; b Department of Cellular and Molecular Nutrition, School of Nutritional Sciences and Dietetics, Tehran University of Medical Sciences, Tehran; c Noncommunicable Diseases Research Center, Endocrinology and Metabolism Population Sciences Institute, Tehran University of Medical Sciences, School of Medicine, Alborz University of Medical Sciences, Baghestan Boulevard, Karaj; d Department of Clinical Nutrition, School of Nutritional Sciences and Dietetics; e Diabetes Research Center, Endocrinology and Metabolism Clinical Sciences Institute, Tehran University of Medical Sciences, Tehran, Iran.

**Keywords:** blood pressure, diabetes, endothelial dysfunction, green cardamom, trial protocol

## Abstract

**Introduction:**

Cardamom possesses antioxidant, anti-inflammation, and blood pressure lowering properties, which might improve endothelial function in type 2 diabetic patients. However, no study has examined the effect of cardamom on diabetic patients. The present study aimed to examine the effects of 10-week green cardamom intake on blood pressure, concentrations of inflammatory and endothelial function biomarkers in type 2 diabetes mellitus patients, and its potential mechanisms.

**Methods and Analysis Design:**

Eighty overweight or obese patients with type 2 diabetes mellitus (aged 30–60 years) will be recruited into the trial and will assign to receive either cardamom (3 g/day, 6 capsules) or placebo (rusk powder, 6 capsules) for a period of 10 weeks. Systolic blood pressure and diastolic blood pressure, asymmetric dimethylarginine, and nitric oxide will be measured. Serum inflammatory markers namely interleukin 6, tumor necrosis factor-α, high-sensitivity C-reactive protein, and factors related to endothelial function including intercellular adhesion molecule-1, vascular cell adhesion molecule 1, CD62 antigen-like family member E, and cluster of differentiation 163 will be measured at baseline and at the end of the trial. Sociodemographic, International Physical Activity Questionnaire, and three 24-hour dietary recall questionnaires will be collected for each participant.

**Ethics and dissemination:**

The study has been approved by The Ethics Committee of Tehran University of Medical Sciences (IR.TUMS.REC.1395.2700). Each participant will sign a written informed consent at the beginning of the study. At the end of the study, results will be published timely manner.

**Trial registration number:**

(http://www.irct.ir, identifier: IRCT-2016042717254N5) Date of registration: 2016-11-23

**Limitations**

- Some patients might not cooperate till the end of the study, which will be replaced with other patients.
- Because of the strong aroma of cardamom the placebo group might find out that they are not taking cardamom and vice versa and this limits the study to be double blinded.
- Small sample size which might affect generalizability of the results.
- Short follow-up period (10 weeks) and dose of the cardamom supplementation (3 g/day).

**Strengths**

- According to our knowledge and searches we have done, this is one of the first randomized controlled clinical trials to assess the effects of *Elettaria cardamomum* supplementation in patients with type 2 diabetes mellitus.
- The present study will provide the required base for future greater clinical trials.
- This is a double-blind study with the least dropout.
- We will place placebo capsules next to cardamom capsules so the placebo capsules will have the aroma of cardamom and by this we try to lessen the limitation of the study.
- We will exclude participants who are taking supplements, as well as those participating in a weight loss program, and thus will be able to exclude potential confounding by these factors.

## 1 Introduction

Type 2 diabetes mellitus (T2DM) is one of the most important causes of mortality and morbidity around the world.^[1]^ The International Diabetes Federation estimates that in 2010, 285 million people of the world population suffered from diabetes and by 2030 this number will increase to 439 million people of the world population.^[2]^ The incidence of cardiovascular diseases (CVDs) is 2- to 4-fold in people with T2DM.^[3]^ CVD is the major reason of morbidity in patients with T2DM, and a cause of death in approximately 3 out of 4 diabetic patients.^[4]^ Raised blood pressure is more common in people with T2DM.^[5]^

CVD is generally attributed to the adverse effects of hyperglycemia and oxidative stress on vascular biology. Vascular endothelial cells play an important role in maintaining cardiovascular homeostasis and endothelial dysfunction causes the development of atherosclerosis. In diabetic patients, endothelial dysfunction is a consistent finding.^[6]^

Inflammation is an integral part of the atherosclerotic process. Plasma concentration of inflammatory mediators, such as tumor necrosis factor-α (TNF-α), interleukin-6 (IL-6), and C-reactive protein (CRP) are higher in obesity and patients with T2DM in which insulin resistance occurs.^[7]^ Inflammatory markers stimulate endothelial production of adhesion molecules such as intercellular adhesion molecules (ICAMs), vascular cell adhesion molecule (VCAM), and endothelial-leukocyte adhesion molecule (E-selectin). These proinflammatory cytokines have a direct effect on vascular walls, which promotes atherosclerosis and leads to impaired vascular reactivity.^[8]^

Cluster of differentiation 163 (CD163) is a macrophage-specific receptor involved in the clearance and endocytosis of hemoglobin-haptoglobin complexes.^[9]^ A high CD163 expression in macrophages is a characteristic of tissues responding to inflammation and indirectly contributes to the anti-inflammatory response. sCD163 is a biomarker for inflammation in adipose tissues and also a biomarker for the development of T2DM.^[10]^ It has been indirectly associated with anti-inflammatory and atheroprotective activity^[11]^ and is a therapeutic target.^[10]^

The exact mechanisms of endothelial dysfunction in T2DM is not known but it might be related to decreased synthesis of nitric oxide (NO) or increased inactivation of NO.^[12]^ NO has cardioprotective roles which include regulation of blood pressure and vascular tone, inhibition of platelet aggregation and leukocyte adhesion, and smooth muscle cell proliferation prevention.^[13]^ NO impairment is regarded as an early step in the development of insulin resistance, atherosclerosis, and T2DM.^[14]^ Asymmetric dimethylarginine (ADMA) is a compound found in human plasma and is endogenous inhibitor of NO synthase.^[14]^ Elevated plasma ADMA levels have been observed in patients with insulin resistance and diabetes, and have been reported to predict adverse cardiovascular events in patients with T2DM.^[15]^

It has been shown that many risk factors for CVD such as hypercholesterolaemia, obesity, and T2DM are conditions in which the activation of the sirtuins had shown to have protective effects in experimental models.^[16]^ Sirtuins are the class III histone deacetylases, which are widely distributed in the body and regulate physiopathological processes, such as inflammation and have cardioprotective effects.^[16,17]^ The best characterized and well-studied among the human sirtuins is sirtuin 1 (SIRT1) which has anti-inflammatory functions in macrophages and endothelial cells. SIRT1 interferes with the transcription factor nuclear factor kappa B (NF-κB) signaling pathway which leads to downregulation of the expression of various proinflammatory cytokines.^[18]^ The production of proinflammatory cytokines in human atherosclerotic plaques is NF-κB dependent^[19]^ and NF-κB activation leads to the expression of many proinflammatory cytokines (e.g., IL-1, IL-2, IL-6, and TNF-α), adhesion molecules (e.g., VCAM, ICAM, E-selectin), and inducible proinflammatory enzymes (cyclooxygenase-2 and inducible NO synthase), which exacerbate the inflammatory process and make it perpetual.^[20]^ The results of a study demonstrate that SIRT1 plays a fundamental role in regulating endothelial NO by activating NO synthase eNOS, which increases endothelial NO.^[21]^ SIRT1 impairs synthesis and increases metabolism of ADMA.^[22]^

There are developing evidence that shows, because of their biological properties, plant foods polyphenols, may be unique nutraceuticals and supplementary treatments for various aspects of T2DM.^[23]^ Polyphenols have been shown to activate SIRT1 either directly or indirectly in vitro and in vivo. Hence, the activation of SIRT1 by polyphenols would be beneficial in therapeutic intervention of a variety of chronic diseases.^[17]^

Seeds of cardamom (*Elettaria cardamomum*) are from the Zingiberaceae family and are used as the spice ingredient in food. Cardamom contains flavonoids like quercetin, kaempferol, luteolin, and pelargonidin.^[24]^ Key components of essential oil in cardamom [i.e., 1,8-cineol (eucalyptol); beta-pinene; geraniol] by binding to NF-κB, provides anti-inflammatory activity.^[25]^ The effect of *E cardamomum* on some of the cardiovascular risk factors in individuals with stage 1 hypertension was studied. The result showed that *E cardamom* effectively reduces blood pressure, enhances fibrinolysis, and improves antioxidant status.^[26]^

Traditional remedies and as a part of that, herbal medicines are a potential source for the development of new therapeutic medicines to be used in the treatment of various diseases.^[27]^ To the best of our knowledge, no study has yet evaluated the anti-inflammatory and vascular protection effect of cardamom in patients with T2DM. The objective of this study is to investigate the effects of green cardamom supplementation on blood pressure and endothelial function. To show the mechanism of cardamom effect, inflammatory markers, CD163, SIRT1, NO, and ADMA will be measured.

## 2 Study design and objectives

A double-blind randomized clinical trial design is to be used in this study.

### 2.1 Objectives

- Compare the mean SBP and DBP and serum NO and ADMA between the 2 groups and within each group, before and after intervention.
- Compare the mean serum inflammatory factors [TNF-α, IL-6, hs-CRP, CD163] between the 2 groups and within each group, before and after intervention.
- Compare the mean of serum ICAM, VCAM, E-selectin, and SIRT1, between the 2 groups and within each group, before and after intervention.

### 2.2 Methods and analysis design

#### 2.2.1 Study design

**Figure 1:**
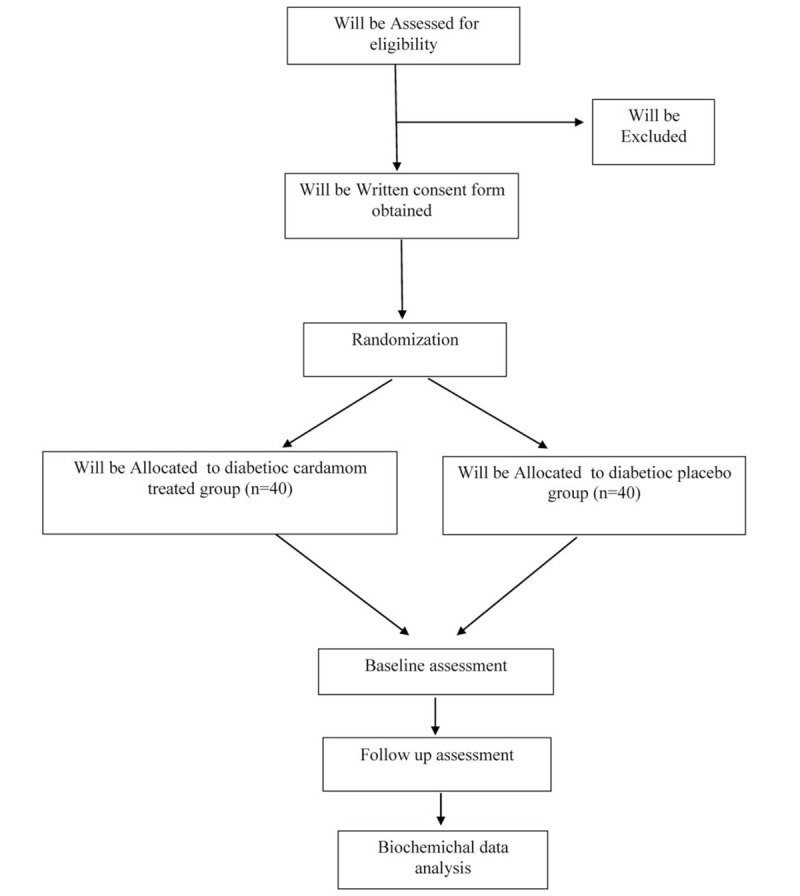
Participants flow diagram.

The study consisted of a parallel, double-blind randomized, placebo controlled clinical trial with treatment and control groups. A flow chart of the study protocol is shown in Figure 1.

#### 2.2.2 Sample size

Eighty patients with T2DM diagnosed by diabetes specialist physicians were recruited from Diabetes Research Center, Endocrinology and Metabolism Clinical Sciences Institute, Tehran University of Medical Sciences Tehran, Iran to detect a significant mean difference of hsCRP,^[28]^ 95% confidence level, and 80% power. Regarding a possible loss to follow-up, a safety margin of 20% was determined The patients were divided equally in the placebo and cardamom supplement groups. We started recruiting participants from Diabetes Research Center from November 20, 2016 to July 20, 2017 and by now we are collecting data from the questionnaires and blood sample tests. 
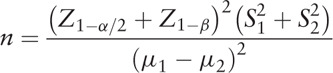


#### 2.2.3 Study population

Patients with T2DM attending the outpatient of Endocrinology and Metabolism Clinical Sciences Institute of Tehran University of Medical Sciences in Tehran will be invited to the study. The patients will be diagnosed by an endocrinologist and by their medical records, using the American Diabetic Association, fasting blood sugar ≥126 mg/dL or 2 hours postparandial ≥200 mg/dL, or HbA1C ≥6.5%.^[29]^ Patients should meet the eligibility criteria. After their identification, the patients will be referred to the investigator to be enrolled in the study and to be explained about the goals, methods, and benefits of the intervention. Written informed consent will be obtained prior to the recruitment. The general questionnaire, 24-hour dietary recall questionnaire, short-form International Physical Activity Questionnaire (IPAQ) and will be completed by interviewers.

#### 2.2.4 Inclusion criteria

Eighty patients within the age group of 30 to 50 years of either sex, body mass index (BMI) between 25 and 34.9 kg/m^2^, patients with T2DM who have been diagnosed with at least 2 years history, according to American Diabetes Association criteria, namely fasting serum glucose >7.0 mmol and last HbA1C value >7%, will be recruited from the regional outpatient diabetes care center. The patients should be on the stable doses of oral antidiabetic drugs throughout the study.

#### 2.2.5 Exclusion criteria

Having any acute illnesses and presence of some chronic diseases including cardiovascular, kidney, liver, and gastrointestinal diseases and taking related drugs and medications. Any**-**inflammatory and thyroid disease; pregnancy and lactation; menopause; being on insulin regimen; individuals taking drugs such as fibrates (which are PPAR-α ligand) and TZD (which are PPAR-gamma ligand); aspirin, warfarin, and antidepressant medication; history of smoking or alcohol intake at least once a week in the past month; changing diet during the past 3 months; consumption of (at least once a week) ginger or other botanical supplements, antioxidant, and multivitamin/mineral supplement in the past 3 months.

#### 2.2.6 Randomization

The participants will be randomly assigned into 2 groups green cardamom powder (n = 40) or rusk powder (n = 40). The permuted block randomization will be used in this study. Stratified randomization will be used to control BMI and gender distribution, those will be 25–29.9/30–34.9 kg/m^2^ for BMI and for gender, respectively. Randomization will be performed by an assistant and the intervention allocation will be blinded as A and B packages for investigators and patients.

#### 2.2.7 Intervention

The participants will be randomly assigned into 2 groups (green cardamom powder or rusk powder) for 10 weeks. Each patient will receive 2 capsules with each meal (3 g/day) for 10 weeks. Every 5 weeks supplements taken will be checked and empty packs will be received and new packages will be delivered to patients and the number of supplements will be counted. The supplements will be given simultaneously with the usual diabetes care drugs that include maintenance of the oral antidiabetic drugs dosage. All patients receive medical nutrition therapy and physical activity enforcement at this clinic. The 24 hour dietary recall will be taken from all patients at the beginning, middle and end of intervention to be confidant of constant dietary intake. Cardamom and placebo capsules will be prepared by Traditional Medicine Research Center, Tehran, Iran. Each capsule contains 0.5 g of whole green cardamom or rusk powder. Placebo capsules will be completely similar to cardamom capsules by the means of shape, size, and color. All capsules containing placebo will be put on the same bag of the cardamom capsules to take the smell of cardamom. Participants will be asked to keep their usual lifestyle including medical nutrition therapy and physical activity level.

Voucher number of green cardamom is *E cardamomum* (L) Maton, Family: Zingiberaceae, PMP-669 which was identified by School of Pharmacy, Tehran University of Medical Sciences. Essential oil content, essential oil compounds including phenolic and flavonoid content of whole green cardamom using GC-mass and HPLC will be measured in the institute of Medicinal Plants, Shahid Beheshti University of Medical Sciences. Some polyphenolic compounds of green cardamom such as caffeic acid, gallic acid, quercetin, and luteolin, which was mentioned in the other article will be determined by HPLC.^[30]^

#### 2.2.8 Adherence

To check patients’ compliance during the 10 weeks, the researcher will interview with them weekly by telephone. In both group (cardamom and placebo), patients will be interviewed to discern whether they are consuming supplements.

#### 2.2.9 Patient safety

Patients will be monitored weekly during the study period and all the occurrence of the adverse events will be assessed by patient interview.

#### 2.2.10 Study outcomes

##### 2.2.10.1 Primary outcome

Primary outcome of this trial includes changes in SBP and DBP, NO, and ADMA. Changes in serum inflammatory markers Il-6, TNF-α, hsCRP, and changes in factors related to endothelial function serum ICAM, VCAM, E-selectin, CD163, and SIRT1.

##### 2.2.10.2 Secondary outcomes

The secondary outcomes of this clinical trial are changes in weight, BMI, and waist circumferences (WCs).

#### 2.2.11 Procedure

Patients will be asked to attend the diabetes research center on 3 occasions. The first time sociodemographic questionnaire will be completed. Blood test will be taken at the first time and week 10 of the study by trained nurses. Twenty milliliter blood sample will be taken after an overnight fasting from the antecubital vein with patients in seated position. Prechilled tubes will be used to collect bloods. Blood sample that are collected will be centrifuged at 3000 rpm for 10 minutes at 4°C to obtain the serum and plasma. Blood sample that are collected will be stored at −80°C until further analysis. Serum inflammatory factors (IL-6, TNF-α, hsCRP, CD163); adhesive molecules (ICAM, VCAM, E-selectin); and ADMA, NO, and SIRT1 will be measured by using the specific kits and methods. Anthropometric measurements including weight, height, and WC will be performed by standard methods. Height will be measured using SECA stadiometer without shoes to the nearest 0.1 cm. Body weight will be measured without shoes and light clothing by using a SECA electronic scale (Seca, Hamburg, Germany) to the nearest 0.1 kg. WC in centimeter will be assessed midway between the lowest rib and the iliac crest using a nonstretchable tape measure. BMI will be calculated by dividing weight by the square of height. Twenty-four hours dietary recall questionnaire and IPAQ will also be completed.

#### 2.2.12 Dietary intake assessment

Dietary intake will be estimated by using three 24-hour dietary recall at the beginning, middle, and at the end of the study. Patients will be asked to recall foods and drinks they consumed 24 hours before the interview by the investigator. Detailed descriptions such as time of the day, food ingredients, and portion size will be obtained by using Pictures of foods commonly consumed in Iran and common household measurement tools. Dietary intake will be analyzed with Nutritionist software version 4 (First Data Bank, San Bruno, CA).

#### 2.2.13 Physical activity levels assessment

IPAQ will be administered to assess the physical activity level of the subjects. The IPAQ-SF form records 3 types of activities and time spent sitting. According to the IPAQ scoring protocol responses will be converted to metabolic equivalent task minutes per week (MET-min/wk): total minutes over the last 7 days spent on vigorous activity, moderate-intensity activity, and walking were multiplied by the energy cost of each activity: 8.0, 4.0, and 3.3 MET for vigorous, moderate, and walking, respectively.^[31]^

Figure 1 presents the overall contents of enrollment, interventions, and assessments. Moreover, the SPIRIT checklist is provided as an additional file.

The trial conduct is to be frequently audited by an assistant though an independent process.

#### 2.2.14 Statistical analysis

The normality of the distribution of data will be assessed by using Shapiro-Wilk tests carried out on each parameter before analysis. By using “regression imputation” missing values will be replaced from a regression equation from observed data. By using intention-to-treat all randomized participants will be analyzed in their group. Independent *t* test will be used to evaluate the differences between the means of 2 independent groups with normal data and Mann-Whitney for non-normal data. In order to assess time-by-treatment interactions and time effects on all outcomes two way repeated-measures regression analysis will be used. A *P* value of <.05 will be considered as significant. All statistical analyses will be performed using SPSS version 21 (SPSS Inc, Chicago, IL).

### 2.3 Data accessibility

The principal investigator will have access to the final trial dataset, and such access for other investigators is limited. The trial results will be presented only through the publication.

### 2.4 Ethical considerations

This clinical trial is conducted after approval of the Ethics Committee of Tehran University of Medical Sciences and completion the informed consent. Whenever a person is unable to continue supplementation he/she will be excluded from the study.

## 3 Discussion

Diabetes is a group of metabolic disorders in which over a prolonged period there are high blood sugar levels which results from cells failure to respond to insulin properly which is due to defects in insulin secretion, insulin action, or both.^[32]^ Obesity and T2DM are linked with a low-grade inflammation state. Inflammation markers are associated with an increased cardiovascular risk.^[33]^ In patients with diabetes, endothelial dysfunction appears to be a consistent finding.^[34]^

Seeds of cardamom (*E cardamomum*) from family Zingiberaceae are used as the spice ingredient in food. Cardamom contains flavonoids such as quercetin, kaempferol, luteolin, and pelargonidin.^[24]^ Epidemiological studies have shown an inverse correlation between polyphenols-enriched diet and reduced risks of CVD. Because of their chemical structures polyphenols may interfere with different factors involved in the pathogenesis of CVD.^[35]^ The present study is performed with the aim of assessing the effects of cardamom powder on inflammation and endothelial function in patients with T2DM.

The strength of this trial is that this study is the first randomized controlled trial to determine the effectiveness of cardamom on the outcome measurements including inflammatory markers, CD163, SIRT1, NO, and ADMA in patients with T2DM.

## Acknowledgments

This research has been supported by Tehran University of Medical Sciences and Health services

## Author contributions

**Data curation:** Ensieh Nasli-Esfahani, Mohadeseh Aghasi.

**Formal analysis:** Mostafa Qorbani, Ensieh Nasli-Esfahani.

**Investigation:** Gity Sotoudeh, Hoorieh Khoshamal.

**Project administration:** Shohreh Ghazi Zahedi, Mohadeseh Aghasi, Hoorieh Khoshamal.

**Supervision:** Gity Sotoudeh, Fariba Koohdani, Fereydoun Siassi, Ali Keshavarz, Mohadeseh Aghasi.

**Writing – original draft:** Shohreh Ghazi Zahedi.

**Writing – review and editing:** Gity Sotoudeh, Shohreh Ghazi Zahedi, Fariba Koohdani, Mohadeseh Aghasi.
